# Consumption Junction: A Case of Peritoneal Tuberculosis-induced Small Bowel Obstruction

**DOI:** 10.5811/cpcem.2017.11.36310

**Published:** 2018-01-11

**Authors:** Michael Poppe, Kelly Peng, Dylan Arnold

**Affiliations:** Naval Medical Center San Diego, Department of Emergency Medicine, San Diego, California

## Abstract

The rapid diagnosis and treatment of tuberculosis (TB) is necessary to prevent the spread of infection to others and reduce morbidity and mortality. Atypical presentations are not often considered in the differential. This patient presented with fever and abdominal pain. Computed tomography of the abdomen and pelvis showed small bowel obstruction, initially attributed to the patient’s Crohn’s disease. Chest radiograph showed diffuse interstitial lung disease, consistent with his diagnosis of sarcoidosis. He had multiple recent negative tuberculin skin tests documented. After being admitted to the surgical service and started on antibiotics, the diagnosis of abdominal TB was discovered following surgical exploration and tissue sampling.

## INTRODUCTION

Tuberculosis remains a worldwide problem, with one third of the earth’s population currently infected.^1^ The incidence of pulmonary tuberculosis (PTB) in the United States has largely diminished over the past century, which has led to fewer U.S. physicians caring for tuberculosis patients. While it may often remain a consideration in the emergency department (ED) for patients presenting with cough and fever or a suspect travel history, it is important to remember that TB can affect any part of the body.

Unlike PTB, the incidence of extrapulmonary tuberculosis (EPTB) has remained relatively constant over the last 15 years, and the proportion of EPTB relative to PTB has increased.^2^,^3^ In EPTB cases, the abdomen can be involved in up to 11% of patients. Bowel obstruction, most commonly secondary to hyperplastic mural thickening, stricture formation or adhesions, is a known potential complication. We present a patient with this complication in the setting of previously diagnosed Crohn’s disease and sarcoidosis.

## CASE REPORT

The patient was a 34-year-old male with a history of Crohn’s disease and sarcoidosis who presented to the ED with two weeks of intermittent fevers, night sweats, and two days of nausea and vomiting. He had intermittent fevers for the prior year. Since beginning his current medication regimen, he had not experienced symptoms from his Crohn’s disease and had baseline mild shortness of breath from sarcoidosis with a dry cough without hemoptysis. He endorsed a five-pound weight loss over the prior month. He stated that he typically had a non-bloody bowel movement daily, but his last bowel movement was two days prior. Review of systems was otherwise negative.

The patient stated that he had taken azathioprine and adalimumab for his Crohn’s disease for the preceding two years, and prednisone and trimethoprim/sulfamethoxazole for his sarcoidosis for the prior six months without any recent dosing changes. His only surgery was a terminal ileum abscess removal and appendectomy three years prior in the Philippines, after which he received his diagnosis of Crohn’s disease. He denied any drug allergies and took no other medications. His only recent travel had been to the Philippines three months prior to visit family. He was up to date on vaccinations and had a negative purified protein derivative (PPD) in the past year.

The only vital sign abnormality was a fever to 101.3 degrees Fahrenheit. Pertinent physical exam findings included anterior cervical chain lymphadenopathy, diffuse mild inspiratory crackles, significant abdominal guarding with rebound tenderness. Bowel sounds were present. Labs showed no significant leukocytosis, normal lactate, a mild microcytic anemia, and a mild acute kidney injury. Erythrocyte sedimentation rate and C-reactive protein were 20 and 8.2 respectively.

**Image 1:**
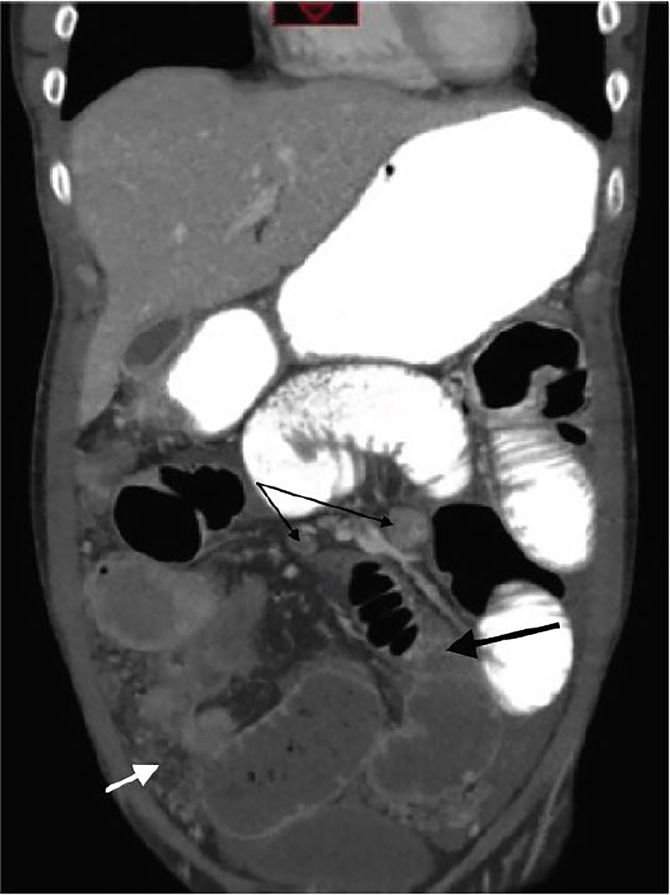
Coronal computed tomography with oral contrast showing oral contrast not completing its path through the bowel (thick black arrow), indicating presence of high-grade obstruction. Also visualized are large mesenteric lymph nodes (thin black arrows), peritoneal studding, and omental caking (white arrow).

Intravenous fluids, antibiotics, and antipyretics were started and a nasogastric tube was placed for what was initially presumed to be a gastrointestinal source of infection with possible obstruction. Abdominal computed tomography (CT) obtained showed signs of intraabdominal infection and obstruction, as seen in Image 1.

**Image 2:**
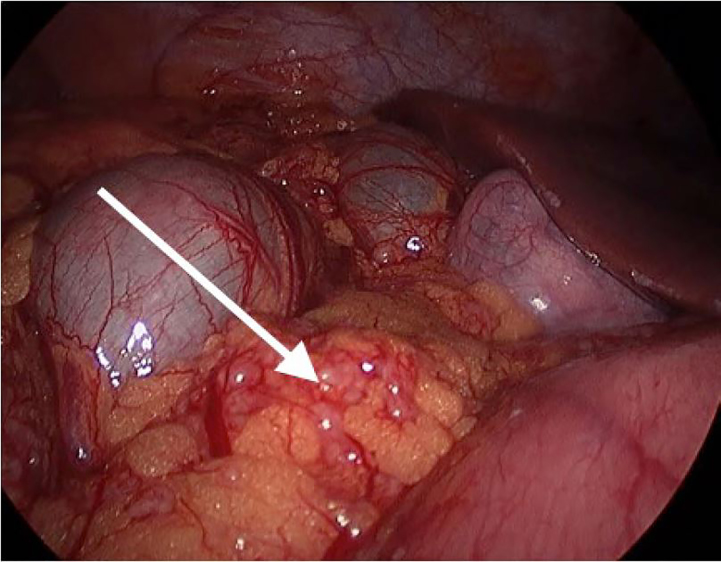
Intra-operative photo showing extensive peritoneal studding (arrow). Histopathology was consistent with a diagnosis of abdominal tuberculosis.

He was admitted to the surgical service. Upon further chart review, the patient was shown to have a positive QuantiFERON® test in 2014 while taking adalimumab and azathioprine, which was never addressed. A colonoscopy was performed to attempt biopsy of the affected tissue, but could not pass through the terminal ileum due to the high-grade obstruction. An exploratory laparotomy was subsequently performed to obtain a definitive diagnosis, as the differential was still broad and included abdominal sarcoidosis, TB, and coccidiomycosis. Histopathological staining was positive for acid-fast bacilli, and the patient was diagnosed and treated for TB. Sputum samples also grew out acid-fast bacilli, indicating concomitant pulmonary infection. Intra-operative photo (Image 2) depicts peritoneal studding, also demonstrated on CT.

CPC-EM Capsule

What do we already know about this clinical entity?

Tuberculosis (TB) is a disease that most commonly affects the lungs, but it can seed nearly any other part of the body.

What makes this presentation of disease reportable?

This is a strong reminder that TB can occur outside of the lungs, can lead to bowel obstruction, and that certain medications increase risk of activation.

What is the major learning point?

TB is not only a disease of the lungs.

How might this improve emergency medicine practice?

Keeping TB in the differential can prevent significant patient and staff morbidity and mortality through early identification and isolation.

Typically, EPTB can be treated with the same drug regimen as pulmonary TB.^4^ However, this patient’s NPO status due to his bowel obstruction necessitated IV therapy. Infectious disease was consulted, and the patient was placed on streptomycin, rifampin, and moxifloxacin until his obstruction resolved. Five days into his hospital course, the obstruction had resolved and the patient was started on oral rifampin, isoniazid, pyrazinamide and ethambutol. His adalimumab and azathioprine were held on admission, and the decision was made to keep holding until his next outpatient visit. He responded well to the drug course, and after a nine-day hospital stay he was allowed to leave after two consecutive sputum cultures were negative.

Approximately three weeks after discharge, the patient had elevated liver enzymes and his pyrazinamide was changed to moxifloxacin. He remained on a four-drug regimen for 10 weeks with significant clinical improvement, and was then transitioned to two-drug therapy with rifampin and isoniazid. He remained on this regimen for eight months. After completing his course, the only residual effect was a mild peripheral neuropathy. He was able to transition back onto adalimumab and azathioprine, and steroid treatment was tapered off successfully.

## DISCUSSION

As demonstrated in this case, abdominal TB can present very similarly to Crohn’s disease.^5^ It is difficult to determine at what point this patient contracted TB; his positive QuantiFERON® in 2014 indicates he had at least been exposed at that time. However, there were no changes made to his drug regimen. The patient remained on adalimumab as well as azathioprine and steroids, which can all decrease sensitivity of the tuberculin skin test, as well as increase risk of activation of latent TB.^6^,^7^,^8^

The abdomen is the most common site for TB to occur after the lungs.^9^ Although uncommon in the U.S., bowel obstruction secondary to abdominal TB is a well-documented issue in countries with higher disease prevalence.^10^ Seeding of the abdomen can occur through hematogenous spread from primary lung foci, lymphatic spread from infected nodes, ingestion of the organism from infected sources (milk products, sputum, etc.), or spread from adjacent infected organ.^11^ A clue on this patient’s imaging was the presence of peritoneal studding and omental caking, which are not typically seen with inflammatory bowel diseases, but can be caused by TB infection in this area.^12^

The patient had been diagnosed with sarcoidosis a few years prior after bronchoalveolar lavage (BAL) revealed non-caseating granulomatous disease without acid-fast bacilli present. On repeat BAL during his hospital stay he grew acid-fast bacilli, and sputum cultures were positive as well. It seems likely that the primary infection in this patient was the lungs, with subsequent spread to the abdominal cavity by one of the previously mentioned pathways.

## CONCLUSION

Diagnosing abdominal TB can be challenging. The disease process is often sub-acute, and the patient presenting with abdominal pain and fever has a large differential. In particular, this condition presents very similarly to inflammatory bowel disease and often requires histological analysis to obtain a definitive diagnosis. Patients may, as in this case, already carry diagnoses that can complicate the picture. Care should be taken in the ED to consider TB in the setting of the patient’s symptoms, comorbidities, immune-suppressing medications, recent travel and exposure history.
